# Mutation Analysis of the Mitochondrial tRNA Genes in Iranian Coronary Atherosclerosis Patients

**Published:** 2017-10

**Authors:** Mohammad Mehdi HEIDARI, Mahboobe DERAKHSHANI, Fatemeh SEDIGHI, Seyed Khalil FORUZAN-NIA

**Affiliations:** 1. Dept. of Biology, Faculty of Science, Yazd University, Yazd, Iran; 2. Dept. of Cardiac Surgery, Afshar Hospital, Shahid Sadoughi University of Medical Sciences, Yazd, Iran

**Keywords:** Atherosclerosis, Mitochondrial tRNA, Mutation, PCR-SSCP

## Abstract

**Background:**

Atherosclerosis is a disease that affects large and medium size arteries in the body that underlies coronary heart disease. Several nucleotide changes in mitochondrial tRNA genes have been reported in various diseases. The purpose of the study was to identify hotspot mitochondrial tRNA mutations in atherosclerotic patients.

**Methods:**

In this case-control study, the variations of ten mitochondrial tRNA genes (about 50%) were investigated in 70 patients from October 2013 and June 2015 suffered from atherosclerosis. The related mitochondrial area was amplified using PCR methid. The mutation analysis was performed by Single Strand Conformational Polymorphism (SSCP) and Restriction Fragment Length Polymorphism (RFLP). All the positive samples were sequenced.

**Results:**

We found one novel heteroplasmic mutation (m.5725T>G) and three reported single nucleotide polymorphisms (SNPs) previously in other diseases including m.5568A>G, m.5711A>G and m.12308A>G.

**Conclusion:**

These tRNA mutations can alter their steady state level and affect the structure of tRNA. The role of mitochondrial tRNA mutations in the pathogenesis of atherosclerosis could potentially be important for the understanding of mitochondrial dysfunction in coronary atherosclerotic plaque formation.

## Introduction

Atherosclerosis is the most common large arteries disease and is still the leading cause of death in the world (1, 2). Reactive oxygen species (ROS), in particular, superoxide and hydrogen peroxide (H_2_O_2_) have been implicated in every aspect of atherosclerosis from its origins as fatty streaks to plaque instability and rupture (3, 4). Considerable evidence support changes in energy metabolism including defective fatty acid oxidation that is one of pathogenic processes involved in atherosclerosis (5).

The main parts of cellular energy in the form of ATP are produced by oxidative phosphorylation process in the mitochondrial respiratory chain enzyme complexes (6). Mitochondrial genome exhibits high nucleotide changes and mutation fixation rates (7). The clinical phenotype of monogenic and multigenic disease in individuals is modulated by modifier genes (8, 9).

The human mitochondrial genome encodes 22 tRNA genes while amino acids of leucine and serine have two tRNA (tRNA^Leu^ reads UUR and CUN codons, and tRNA^Ser^ reads UCN and AGY codons) (8, 10). Therefore, the importance of each of these individual tRNAs in mitochondrial protein synthesis is obvious (11). Most mitochondrial nucleotide changes lead to polymorphisms and some are considered pathogenic (12).

There is no study investigating the role of mtDNA mutations in atherosclerosis in Iranian population, so we evaluated about 50% mitochondrial tRNA genes including hotspot mitochondrial tRNA mutations by PCR-SSCP and PCR-RFLP and automated DNA sequencing in atherosclerotic patients.

## Materials and Methods

### Patients

In this case-control study, we studied seventy Iranian patients (30 females and 40 males) from unrelated families previously documented by coronary angiography from October 2013 and June 2015 in the Especial Afshar Hospital (Yazd, Iran). The carotid arteries imaging was performed by ultrasonography to assess the extent of carotid atherosclerosis. The protocol of ultrasound examination involved the scanning of the right and left common carotid artery and the area of the carotid sinus (bulb) as high up as possible (13).

Coronary arterial disease (CAD) was considered present when up to 50% blockage, induced by stenotic lesions, was observed in the major epicardial coronaries and their branches (Table 1). Subjects’ normal, formed the control group. We also chose 65 healthy controls that matched for age, sex, and ethnicity.

**Table 1:** The Summary of the clinical of coronary atherosclerosis patients

***Variable***	***Patients (n=70)***	***Controls (n=65)***	***P-value***
Male gender (%)	57	53	0.83
Age, years	54.1±7.3	52.1± 7.4	0.49
Smokers (%)	25.7	18.7	0.24
Body mass index (kg/m^2^)	24.3±2.1	23.2±1.9	0.028
Cholesterol, mg/dl	211.3±51.4	170.2±35.6	0.001
LDL-C, mg/dl	125.7±43.7	113.7±46	0.122
HDL-C, mg/dl	45.6±8.3	47.7±11.9	0.233
TGs, mg/dl	200.3±104.1	157.8±95.6	0.015

The study was approved by the research Ethics Committee of the Yazd University. All of the patients and the control group were informed of the aims of the study. They gave their informed consents for the genetic analysis.

### Mutation analysis by SSCP

DNA was isolated from the peripheral blood samples using a DNA extraction kit (DNAfast-Kit-Genfanavaran, Tehran, Iran). Mitochondrial tRNA^Asn, Tyr, Cys^, tRNA^Ala, Trp, Asp^ and tRNA^Ile, Gln, Met^ genes were amplified by PCR and assayed by SSCP. The primers used in PCR, the sizes of PCR products are listed in Table 2. PCR was performed in a total volume of 25 μL containing 100 ng of template DNA, 10 pmol of each primer, 200 μM dNTPs, 2.5 mM MgCl2, 1X buffer, and 1 U of *Taq* polymerase (Sinnaclone, Tehran, Iran). PCR amplification was carried out at 94 °C for 5 min, followed by 35 cycles of denaturation at 94°C for 30 sec, annealing touchdown from 64 °C to 55 °C (14 cycles) with remaining cycles at 50 °C for 35 sec, and extension at 72 °C for 30 sec, followed by a final extension for 5 minutes. After electrophoresis, gel was stained with the silver staining method.

**Table 2:** Primers used for mtDNA amplification

***Primer sequence (5′-3′)***	***Primer position***	***Tm (°C)***	***Size (bp)***	***Gene***
F: GCAGGGACCAACGAATGCT	4211–4230	59	330	tRNA^Ile, Gln, Met^
R: CCTTCCTCACACTGGCACTTGTA	4540–4521			
F: CCCTTACCACGCTACTCCTA	5461–5480	59	280	tRNA^Ala, Trp, Asp^
R: GGCGGGAGAAGTAGATTGAA	5740–5721			
F: CAAACACTTAGTTAACAGCT	5681–5700	59.5	300	tRNA^Tyr, Cys, Asn^
R: GCTCATGCGCCGAATAG	5980–5961			
F: ATTAATTCCCCTAAAAATCT	8221–8240	57	250	tRNA^Lys^
R: TAGGTGGTAGTTTGTGTTTA	8470–8451			

For SSCP assay, PCR products were heat-denatured at 93 °C for 3 min and chilled on ice for 3 min, and loaded onto a polyacrylamide/TBE 0.5x gel containing. The gel concentrations and running conditions were as follows: 8% polyacrylamide gel, 16h, room temperature, 200 V. After the run, the gel was removed from the apparatus and the DNA bands were visualized through silver staining.

### Genotyping of mitochondrial 12308 polymorphism in tRNA^Leu(CUN)^

Mitochondrial m.12308A>G polymorphism were detected using primers that introduce a restriction site into the wild-type nucleotide sequences by mispairing-PCR (14). This PCR contains a nucleotide that differs from the Cambridge mtDNA sequence (15) (A to G substitution). A 144 bp fragment encompassing the tRNA^Leu(CUN)^ mutation site, located at nt 12308, was amplified by mispairing PCR. With this modification, an EcoRI site is created. The mtDNA fragment was digested with EcoRI and the fragments (119 and 25 bp) were produced.

### Statistical analysis

Chi-square and Fisher’s exact tests were used for comparison of categorical variables. This Statistical analysis was performed by the SPSS version 20 (SPSS Inc., Chicago, Il, USA) and *P*<0.05 were regarded as statistically significant.

## Results

SSCP analyses for the mitochondrial tRNA gene were conducted on a total of 70 patients and 65 healthy controls. Mean age (mean ± SD) was 54.1±7.3 and 52.1± 7.4 yr for patients and controls, respectively. Coronary angiography revealed 70 patients (CAD^+^ group) with one-vessel (LAD) (n=18), two-vessels (LCX) (n=26), or three-vessels (RCA) (n=26) that were candidate for CABA (Coronary Artery Bypass Graft) and 65 patients (CAD^−^ group) with no angiographically identified narrowing.

**Fig. 1:**
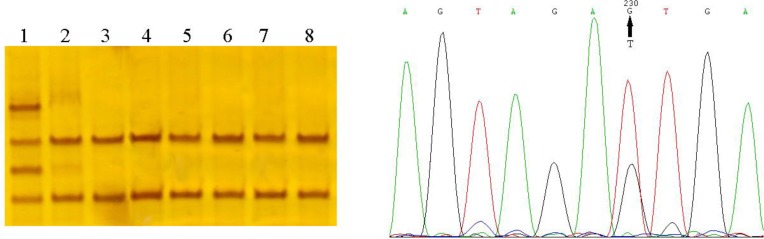
Identification of a HeteroplasmicmtDNA mutation in CAD patient by SSCP and sequencing. Lane 1, Heteroplasmic band shift belong to CAD patient. Lane 2–8, wild type. Sequencing result revealed T5725G mutation.

We found three reported nucleotide variations in thirty-one patients and did not find any of these mutations in the healthy controls. These variations include m.5711A>G transition (homoplasmic state) in D-loop of tRNA^Asn^ gene, m.5725T>G transversion (heteroplasmic state) in acc. stem of tRNA^Asn^ gene (Fig. 1) and m.12308A>G polymorphism in tRNA^Leu(CUN)^ gene and one mutation include m.5568A>G transition (homoplasmic state) in tRNA^Trp^ in T-loop (Table 3). The m.12308A>G polymorphism was found in 24 (34.2%) patients and 11 (16.9%) normal controls (Table 4). In the patient’s group, m.12308A>G polymorphism was detected 7 (29%) heteroplasmic and 17 (71.1%) homoplasmic.

**Table 3:** Mitochondrial variations found in coronary atherosclerosis patients

***Locus***	***Gene***	***Position***	***Sequence change***	***No. of Patients***	***Hetero/Hoo***	***Previously reported (MITOMAP web Site)***
MT-TW	tRNA^Trp^	5568	A>G	1	Homo	No
MT-TN	tRNA^Asn^	5711	T>A	4	Hetero	Yes
MT-TN	tRNA^Asn^	5725	A>G	3	Homo	Yes
MT-TL2	tRNA^Leu (CUN)^	12308	A>G	24	Homo/Heteo	Yrs

**Table 4:** Frequencies of the 12308 A>G mutation in the whole study population

***Nucleotide variation***	***CAD^+^ (n=70)***			***CAD^−^ (n=65)***	P-value*
	**Single vessel disease**	**Double vessel disease**	**Triple vessel disease**		
A	12 (17.1 %)	16 (22.9 %)	18 (25.7 %)	54 (83.1 %)	0.03
G	6 (8.6 %)	10 (14.3 %)	8 (11.4 %)	11 (16.9 %)	

* Difference between CAD^+^ and CAD^−^ group

## Discussion

More than 115 mitochondrial pathogenic mutations have been found in the mitochondrial genome (16) with 38% occurring in protein genes (respiratory chain subunits), and 62% in mitochondrial protein synthesis related genes (4% in ribosomal and 58% in tRNA genes). Mitochondrial tRNA genes are conserved evolutionally. More nucleotide changes in tRNA genes are nonpathogenic and are located in no conservational sites. Therefore, the finding of nucleotide changes in conservational tRNA sites might be pathogenic. The mitochondrial genome evolves at a much faster rate than the nuclear genome. The mtDNA mutates at a rate that is 10–17-fold higher than that of the nucleus (17).

Some single mitochondrial mutations (1555A>G, 3256C>T, 12315G>A, and 15059G>A) had a higher prevalence in atherosclerotic tissue, and the proportion of mtDNA copies bearing mutant allele was higher (18). Besides, mutation m.3256C>T in atherosclerosis and coronary heart disease could be used as genetic marker (19).

Five different mutations in the tRNA^Asn^ and five different mutations in tRNA^Trp^ have been described. They were associated with chronic progressive external ophthalmoplegia (CPEO) (20), multiple organ failures (MOF) (21), and progressive external ophthalmoplegia (PEO) (2), maternally inherited Leigh syndrome (MILS) (22). Here we report two point mutations in tRNA^Asn^ and one mutation in tRNA^Trp^. m.5711A>G mutation in tRNA^Asn^ and m.5568A>G mutation in tRNA^Trp^ was homoplasmic and were reported as polymorphism previously (23–25). Six hetetoplasmic mitochondrial DNA variants were identified among diabetic patients with arterial stenosis (26).

m.5725T>G mutation in tRNA^Asn^ was heteroplasmic. This mutation was never reported as a neutral polymorphism and was not detected in normal individuals from different ethnic backgrounds.

**Fig. 2:**
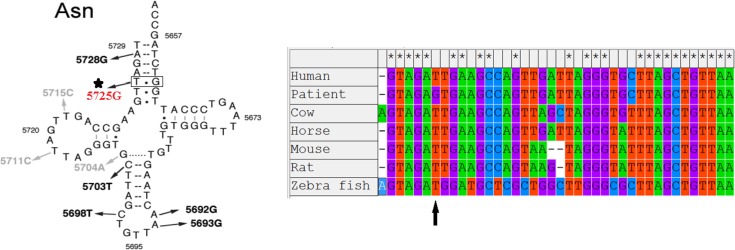
Alignment of 5725T>G of mitochondrial tRNA^Asn^ gene and the arrow indicate the sites of mutation 5725T>G

The m.5725T>G change considers as pathogenic mutation for the subsequent reasons. First, this mutation is located in structurally/functionally important region. Second, this change was never reported as a neutral polymorphism and was not detected in normal individuals from different ethnic backgrounds. Third, the mutation was heteroplasmic in lymphocyte cells analyzed, and heteroplasmic is a common feature of pathogenic mtDNA mutation. Fourth, tRNA^Asn^ sequence and the two-dimensional (2D) structure comparison according to Mamit-tRNA database (http://mamit-trna.u-strasbg.fr/) showed m.5725T>G mutation is highly conserved between species during evolution (Fig. 2).

A mutation was identified in the mitochondrial tRNA^Leu(CUN)^ gene that encodes for the most common amino acid. This mutation previously reported in Wolfram syndrome and Chronic Progressive External Ophtalmoplegia (CPEO) (27). The tRNA^Leu(CUN)^ sequence and the two-dimensional (2D) structure comparison, per the Mamit-tRNA database (http://mamit-tRNA.ustrasbg.fr), showed that the 12308A>G mutation is highly conserved between species. The 12308A>G mutation is found in breast cancer (28), and an increased risk of stroke was associated with the presence of a homoplasmic 12308A>G variant in 48 patients (29). We found significant statistical correlation between the incidence of m.12308A>G polymorphism and atherosclerosis (*P*=0.03) and this mutation was not comparable in patients with single, double, or triple vessel disease (Table 4).

The novel tRNA^Asn^ mutation described here further underlines the role of mitochondrial tRNA mutations as a cause of the pathogenesis of mitochondrial diseases. Therefore, to find out and understand the nature of pathogenesis and predisposition effects of novel variations on atherosclerosis, further genetic and functional studies are necessary.

### Study limitations

No accessibility to tissue samples from our patients is the major limitation of our study. Another limitation is the lack of classification of the patients according to their angiographic findings. Further studies with larger cohorts of patients are warranted to reveal the relationship of mitochondrial nucleotide changes with atherosclerotic risk factors.

## Ethical considerations

Ethical issues (Including plagiarism, informed consent, misconduct, data fabrication and/or falsification, double publication and/or submission, redundancy, etc.) have been completely observed by the authors.
